# A look at the ASEAN-NDI: building a regional health R&D innovation network

**DOI:** 10.1186/2049-9957-3-15

**Published:** 2014-04-28

**Authors:** Jaime C Montoya, Carina L Rebulanan, Nico Angelo C Parungao, Bernadette Ramirez

**Affiliations:** 1 Department of Science and Technology-Philippine Council for Health Research and Development (DOST-PCHRD), Taguig City, Philippines; 2 UNICEF/UNDP/World Bank/WHO Special Programme for Research and Training in Tropical Diseases (TDR), World Health Organization, Geneva, Switzerland

**Keywords:** ASEAN-NDI, Health research and development, Infectious tropical diseases, Innovation network, Strategic business plan

## Abstract

Globally, there are growing efforts to address diseases through the advancement in health research and development (R&D), strengthening of regional cooperation in science and technology (particularly on product discovery and development), and implementation of the World Health Assembly Resolution 61.21 (WHA61.21) on the Global Strategy and Plan of Action on Public Health, Innovation, and Intellectual Property (GSPA-PHI). As such, the Association of Southeast Asian Nations (ASEAN) is responding to this through the establishment of the ASEAN-Network for Drugs, Diagnostics, Vaccines, and Traditional Medicines Innovation (ASEAN-NDI). This is important in the ASEAN considering that infectious tropical diseases remain prevalent, emerging, and reemerging in the region. This paper looks into the evolution of the ASEAN-NDI from its inception in 2009, to how it is at present, and its plans to mitigate public health problems regionally and even globally.

## Multilingual abstracts

Please see Additional file 1 for the translations of the abstract into the six official working languages of the United Nations.

## Review

### The need for an ASEAN-led Health R&D innovation

The Association of Southeast Asian Nations (ASEAN) has a continuum of member nations at different stages of economic and health development, with the majority of the countries belonging to the middle- and low-income categories [1].

While there are ASEAN member states that are more advanced in terms of health systems and programs, the majority still suffer from communicable and non-communicable diseases [2,3]. Infectious tropical diseases also continue to be a challenge to the health sector despite developments in health research and development (R&D) and medical technology. There is widespread concern about the emergence and reemergence of some infectious diseases such as tuberculosis, Severe Acute Respiratory Syndrome (SARS), avian influenza, and Chikungunya [4].

These tropical infectious diseases form part of the neglected tropical diseases (NTDs) alluded to by international organizations. These diseases include infections that have shown low economic returns for investments in health products R&D. However, from a human capital perspective, these so-called NTDs not only place a significant burden on the health system, but they also adversely affect productivity and efficiency of human capital in their respective societies [5].

Budgetary limitations for health research also cause difficulty in pursuing R&D initiatives. With scarce resources for health research, efforts to pursue health research activities in many of the ASEAN nations are limited [5].

In terms of human resources, the ratio of R&D personnel to the population varies across the ASEAN nations with countries including Singapore, Malaysia, and Thailand having more R&D personnel than the rest of the ASEAN nations. The proportion of researchers to population is also much higher in the more developed nations in the ASEAN (e.g., Singapore and Malaysia) compared to those which are less economically developed [6]. It is apparent that many of the ASEAN nations fall below the ratio of researchers to population recommended by the United Nations Educational, Scientific and Cultural Organization (UNESCO). This significantly reduces R&D productivity in the region.

Considering all of these public health challenges and hindrances to providing better quality of health in the ASEAN, activities towards the establishment of a regional health innovation network were initiated with the goal of enhancing product discovery and providing a sustainable essential health R&D through intraregional collaboration—a framework suggested by the World Health Assembly (WHA) Resolution 61.21, Global Strategy and Plan of Action on Public Health, Innovation, and Intellectual Property (GSPA-PHI) [7,8].

### Conceptualization of the ASEAN-NDI

The ASEAN Network for Drugs, Diagnostics, Vaccines, and Traditional Medicines Innovation (ASEAN-NDI) was founded in 2009 in line with the objectives of the GSPA-PHI, which include promotion of R&D, development of North–South and South–South partnerships to support capacity building, and establishment of strategic research networks to facilitate better coordination of stakeholders. It was conceptualized to parallel the African Network for Drugs and Diagnostics Innovation (ANDI), a network championed by the World Health Organization Special Programme for Research and Training in Tropical Diseases (WHO-TDR), which started the idea of establishing regional innovation networks.

**Figure 1:**
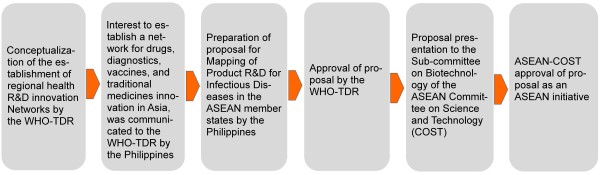
**First stage of the establishment of the ASEAN-NDI.** Following the establishment of the ANDI, interest to establish a similar health R&D innovation network, the ASEAN-NDI, was communicated by the Philippines to the WHO-TDR through PCHRD-DOST. With funding support from the WHO-TDR, the activities of the Network were started, including the ASEAN health R&D landscape mapping.

The ASEAN-NDI is a regional innovation network composed of the ASEAN member states, namely: Brunei Darussalam, Cambodia, Indonesia, Lao PDR, Malaysia, Myanmar, Philippines, Singapore, Thailand, and Vietnam. Its concept was proposed by the Philippines to the ASEAN and was first discussed among the ASEAN member states during the 40^th^ Meeting of the ASEAN Sub-Committee on Biotechnology (SCB) in Bali, Indonesia on 25–26 May 2009, and was later adopted by the ASEAN Committee on Science and Technology (COST) as its own initiative. The ASEAN, through the COST, approved the creation of the ASEAN-NDI in 2009. Start-up funds to support the establishment of the Network were provided by the WHO-TDR. The Philippine Council for Health Research and Development (PCHRD) of the Department of Science and Technology (DOST) served as the secretariat of the ASEAN-NDI, with its Executive Director, Dr. Jaime Montoya, as the overall coordinator (see Figure 1).

The ASEAN-NDI was established to ensure that health technology development and the capacity of member states are appropriately maximized and managed according to regional health needs. It aims to build a sustainable partnership among the ten ASEAN countries to rapidly build the needed human resource, technology, and financing for health development and security.

The enhancement of public R&D capacity to prioritize the public good elements of innovative policy tools and harness the private sector is one strategy most relevant to the 61^st^ WHA framework. The aim was to build developing countries’ leadership and capability in addressing major endemic health issues, as well as issues concerning access to health products by the poor. One of the critical elements of the framework was setting up R&D networks in disease endemic countries [6].

The ASEAN-NDI addresses this opportunity directly, answering both the economic reasoning for public action when markets fail, and the moral basis for an intergovernmental response to the lack of access to health products among the poor in these countries.

## The mapping activity

To lay the groundwork towards this initiative, the ASEAN SCB endorsed a mapping activity to be carried out in order to assess the product R&D landscape for the triple burden of disease in the region, including infectious tropical diseases, non-communicable diseases, and preventable diseases due to accidents and traumas.

“Mapping of Product R&D Landscape for Infectious Tropical Diseases in ASEAN Member States” was conceptualized with the aim of mapping out the capabilities of the ASEAN member countries on drugs, diagnostics, vaccines, and traditional medicine innovation on infectious tropical diseases; identifying gaps and opportunities in the ASEAN; creating a database of institutions, networks, and initiatives with capacities for innovation; and providing the template for the establishment of an ASEAN regional network for innovation in product R&D.

The planned mapping activity was further refined during the first organizational meeting held in Manila, Philippines on 21 October 2009. Attended by delegates from the ASEAN who were identified through the assistance of the SCB members and the ASEAN Secretariat, participants agreed to focus the mapping exercise on infectious diseases such as malaria, tuberculosis, schistosomiasis, dengue, leishmaniasis, lymphatic filariasis, and helminthiases, as well as on other diseases of public health importance.

The mapping activity was conducted from December 2009 to November 2010, through survey and key informant interviews among researchers and institutions, and a review of Elsevier’s Scopus database. The mapping activity gathered records on institutional data of R&D institutions across the ASEAN region.

The results of the mapping exercise indicated a diverse spectrum of capacity for the ASEAN member states to pursue R&D on drugs, diagnostics, vaccines, and traditional medicine.

The exercise showed that there is keen interest among the ASEAN institutions in each country to pursue health R&D. While some countries such as Brunei Darussalam and Cambodia are still in their infancy, others such as Singapore, Malaysia, and Thailand are far advanced in R&D. One measure for this is the number of biomedical and infectious disease articles that these three countries produced from 2005–2009 as compared to the other member states (see Table 1).

**Table 1:** Number of articles and ranking of the ASEAN member States with biomedical articles, 2005–2009

**Country**	**Number of biomedical articles**	**Rank**	**Number of articles on infectious diseases**	**Rank**
Thailand	12,568	1	2,698	1
Singapore	12,405	2	578	2
Malaysia	7,071	3	509	3
Indonesia	1,324	4	335	4
Cambodia	318	6	204	5
Philippines	574	5	154	6
Laos	168	7	80	7
Myanmar	103	9	50	8
Brunei Darussalam	163	8	25	9
Vietnam	86	10	25	9

**Figure 2:**
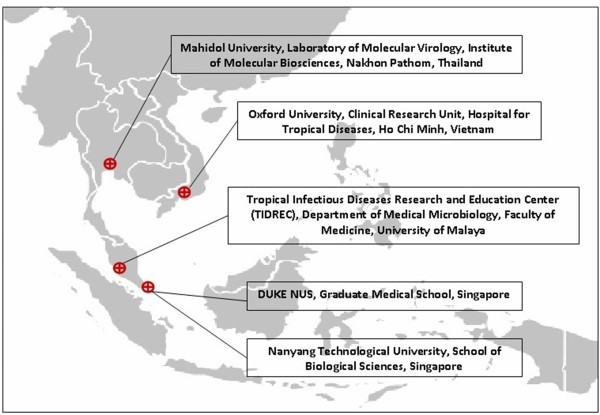
Academic and academy-associated dengue research centers located in the ASEAN.

The ASEAN region has substantial human resources and institutions that can support the pursuit of R&D on drugs, diagnostics, vaccines, and traditional medicine. There are major institutions located in the different ASEAN nations that can be tapped for future collaboration (see Figure 2). The ASEAN also has a number of institutions that have the capacity to produce drugs, diagnostics, vaccines, and traditional medicine (see Table 2).

**Table 2:** Number of institutions involved in drug, diagnostics, vaccines, and traditional medicine development in the ASEAN

***DRUG DEVELOPMENT***							
**Country**	**No. involved in Drug Development**	**No. involved in Target Discovery**	**No. involved in Screening**	**No. involved in Product Development**	**No. involved in Clinical Trials**	**No. involved in Manufacturing**	**No. involved in M&E**
Brunei^a^	-	-	-	-	-	-	-
Cambodia^c^	20	10	-	2	-	10	10
Indonesia^b^	40	22	23	14	18	9	16
Laos^b^	3	-	-	-	-	-	-
Malaysia^b^	33	10	6	7	3	1	3
Myanmar^c^	19	14	3	-	2	-	2
Philippines^b^	19	6	6	3	9	2	7
Singapore^d^	6	6	-	4	1	-	1
Thailand^c^	90	59	-	12	27	-	33
Vietnam^b^	18	4	-	6	6	12	-
***DIAGNOSTICS DEVELOPMENT***							
**Country**	**No. involved in Diagnostics Development**	**No. involved in Target Discovery**	**Platform Development**	**No. involved in Product Development**	**No. involved in Clinical Trials**	**No. involved in Manufacturing**	**No. involved in M&E**
Brunei^a^	-	-	-	-	-	-	-
Cambodia^c^	4	-	-	1	2		2
Indonesia^b^	21	16	1	4	-	2	18
Laos^b^	-	-	-	-	-	-	-
Malaysia^b^	50	15	12	11	6	-	6
Myanmar^c^	2	-	-	-	1	-	1
Philippines^b^	13	11		8	6	2	1
Singapore^e^	5/546	5	4	-	-	-	5
Thailand^c^	31	6	8	3	-	-	15
Vietnam^b^	7	4	5	4	1	2	-
***VACCINES DEVELOPMENT***							
**Country**	**No. involved in Vaccine Development**	**No. involved in Target Discovery**	**No. involved in Screening**	**No. involved in Vaccine Development**	**No. involved in Clinical Trials**	**No. involved in Manufacturing**	**No. involved in M&E**
Brunei^a^	-	-	-	-	-	-	-
Cambodia^b^	14	8	1	-	-	-	1
Indonesia	14	2	-	7	2	2	1
Laos^a^	-	-	-	-	-	-	-
Malaysia^b^	9	4	-	2	2	-	1
Myanmar^b^	9	1	8	-	-	-	-
Philippines^b^	12	6	1	1	5	1	1
Singapore^f^	4/110	4	4	-	-	-	-
Thailand^b^	21	12	4	2	-	-	5
Vietnam^g^	6	-	-	-	-	5	-
***TRADITIONAL MEDICINE DEVELOPMENT***							
**Country**	**No. involved in Traditional Medicine Development**	**No. involved in Target Discovery**	**No. involved in Screening**	**No. involved in Product Development**	**No. involved in Clinical Trials**	**No. involved in Manufacturing**	**No. involved in M&E**
Brunei^a^	-	-	-	-	-	-	-
Cambodia^c^	2	-	-	-	-	-	-
Indonesia^b^	16	7			2	1	2
Laos^b^	-	-	-	-	-		-
Malaysia^b^	188	8	5	6	2	-	2
Myanmar^c^	-	-	-	-	-	-	-
Philippines^c^	16	12	14	8	4	3	1
Singapore^h^	-	-	-	-	-	-	-
Thailand^c^	4	2	-	2	-	-	-
Vietnam^b^	12	5	6	5	3	7	-

**Sources:** Scopus (Elsevier, 2010) and country reports.

^a^No specific information available.

^b^Data based on the country report.

^c^Data from internet sources.

^d^Although based on the country report, this does not summarize the R&D in Singapore.

^e^The country report states five institutions while internet sources indicate as many as 546 institutions.

^f^The country report states four institutions while the internet source cites 110 institutions.

^g^The involvement of institutions in the specific stage of development is not available in the supplied information.

^h^No data available for Singapore.

One of the challenges that arises from the disparity across the ASEAN countries is that a number of countries work on the same disease and for the same product (i.e., drugs, vaccines, etc.), which results in duplication and wastes time and resources. This shows that although collaborating ventures may have limitations imposed by funding/sponsoring partners, the ASEAN member states need to explore and expand collaborative engagements to other ASEAN member states with the end goal of enhancing capacity in the region and minimizing duplication of efforts.

**Figure 3:**
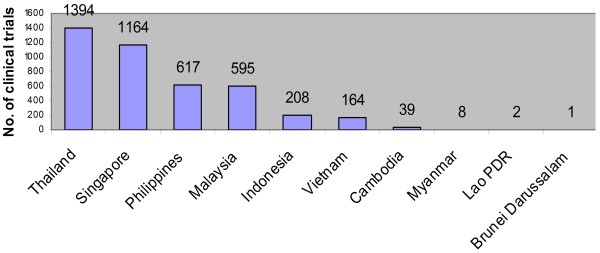
**Number of Clinical Trials conducted per country in Southeast Asia.** Countries in the ASEAN region have been recently identified as an emerging potential in the field of clinical trials. In order to assess the focus of current clinical trials in the region, absolute numbers of clinical trials were obtained from the Clinicaltrials.gov database (as of July 2010). It was found that most clinical trials reported are focused on maladies and conditions such as neoplasms and cancers [9].

Asia, including the ASEAN, is a major player in the future of pharmaceuticals. The recent slowdown in major Western countries has led to the outsourcing of developmental work in developing countries including the ASEAN (see Figure 3). The growing presence of clinical research organizations in the region is a testimony to the presence of capable human resources and growing importance of the region in the future of the pharmaceutical industry.

The disparity across the ASEAN member states indicates some gaps that need to be addressed. There is a problem with technology know-how and priority funding, especially among countries that are dependent on imported pharmaceuticals. There is a problem of infrastructure and resources in some countries, which can be addressed by implementing working arrangements among the ASEAN member states, and by sharing resources and expertise. Concerns such as funding and logistics support, and ethical considerations need to be addressed by their respective countries and by the ASEAN as a whole. Intellectual property concerns, which may arise from collaborative ventures, are issues that need to be laid down and agreed upon at the onset.

**Figure 4:**
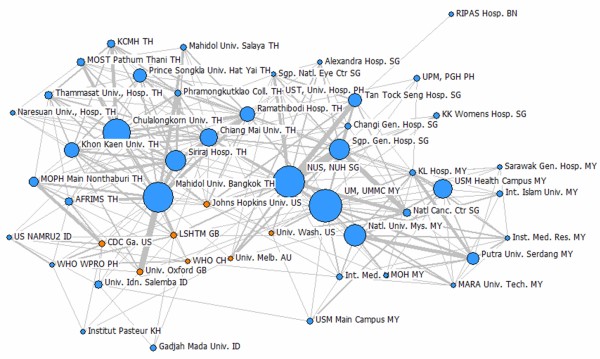
**Networks for diagnostics research collaborations among the top 50 most productive institutions (within and outside the ASEAN) based on articles on diagnostics.** Size of the nodes indicates relative number of articles. Thicker links indicate more instances of collaboration between the two institutions. Blue nodes are institutions in the ASEAN, while orange nodes represent institutions outside the ASEAN. Thai, Singaporean, and Malaysian universities and research centers have most of the collaborations on publications on diagnostics compared with the other ASEAN countries. Most of the collaborations are with the ASEAN member states but there are some collaborations with research centers in the United States, Great Britain, Australia, and Switzerland [11].

**Figure 5:**
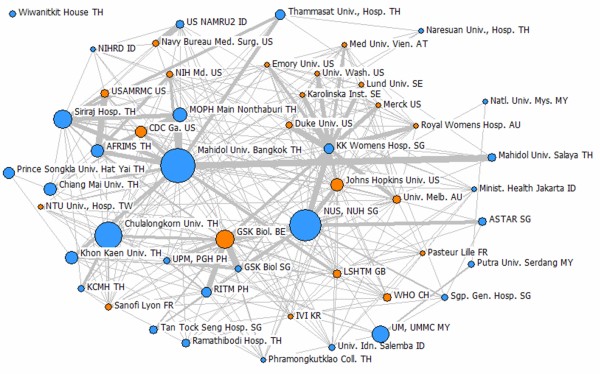
**Network for vaccine research collaborations among the top 50 most productive institutions (within and outside the ASEAN) based on articles on vaccines.** Thai, Malaysian, and Singapore-based research centers dominate the scene with their networks in vaccine development. The collaborations are not only within the ASEAN but also with other countries such as the USA, Australia, Belgium, Austria, France, South Korea, Taiwan, Sweden, and Switzerland. GSK Belgium was a dominant collaborating partner in the ASEAN with respect to vaccine-related articles [11].

The extensive collaboration among the ASEAN member states and with other major research centers in the world indicates that the ASEAN countries have the capacity to pursue collaborative R&D activities on health products development (see Figures 4 and 5). The ASEAN has been used in several free trade agreements and harmonization initiatives. Hence, embarking on an ASEAN initiative that will strengthen the scientific ties to address neglected diseases will not be difficult. The ASEAN member states can facilitate the exchange of expertise, resources, and health products as they have been working in other initiatives to improve the region. Under the ASEAN umbrella where harmonization and collaboration are the key elements, these arrangements can be easily addressed [10].

The results of the mapping were presented and discussed during the second organizational meeting held in Manila, Philippines on 6 December 2010. A website, http://www.asean-ndi.org, was created to serve as a repository of the mapping exercise-related information.

### The Strategic Business Plan (SBP)

The conclusions from the mapping activity triggered the call for the preparation of a Strategic Business Plan (SBP) and the creation of a Task Force (TF) that will serve as an ad hoc group who will guide the development of the plan. On 20 October 2011, the first ASEAN-NDI Task Force meeting was held in Manila, Philippines where the initial outline of the draft SBP and timelines were discussed.

The ASEAN-NDI is envisioned to become Asia’s premier facilitator for collaborative innovation in R&D for health products, benefiting primarily the ASEAN but more open to global markets. As a network involving the ten ASEAN member countries, the ASEAN-NDI will build the needed *human resources, technological capacity, and financing to ensure sustainable health, development, and security*. These inputs will be translated into research and eventually into the production of innovative health products and services that will be made available within and even outside the ASEAN. The vision, mission, and objectives of the ASEAN-NDI are summarized in Table 3.

**Table 3:** The ASEAN-NDI’s vision, mission, objectives, and key stakeholders

**Vision**	To be Asia’s premier facilitator for collaborative innovation in research and development in health products.
**Mission**	To address the unmet public health needs of the ASEAN nations through the advancement of the ASEAN-led health product innovation in the areas of drugs, vaccines, and diagnostics in order to improve health outcomes in the ASEAN region and beyond, and to support sustainable regional economic development.
**Objectives**	• To ensure that health technology development and the capacity of member states are appropriately maximized and managed according to regional health needs.
	• To build a harmonious and sustainable partnership among ASEAN countries and networks to rapidly build up the needed human resource, technology, and financing for health and development security.
	• To capacitate ASEAN member states and help them provide health products and services for their own needs and the needs of the ASEAN as a whole especially in addressing diseases endemic in the region.
	• To contribute to the “ASEAN Community 2015” initiative of the region, in terms of health R&D cooperation
**Key stakeholders**	The success of the establishment and operationalization of the ASEAN-NDI requires the perspective and engagement of key stakeholders from the public, private, and non-profit sectors at every step of the process.
	Such stakeholders include the various ASEAN national governments and their respective science and technology, and health ministries; public and private ASEAN research institutions and researchers; pharmaceutical, medical device, and other health product companies and manufacturers; and potential partner agencies such as the Asian Development Bank (ADB) and the WHO; international organizations and non-governmental organizations; the ASEAN nationals in the diaspora; and any other parties interested in supporting research and development in the ASEAN region.

**Figure 6:**
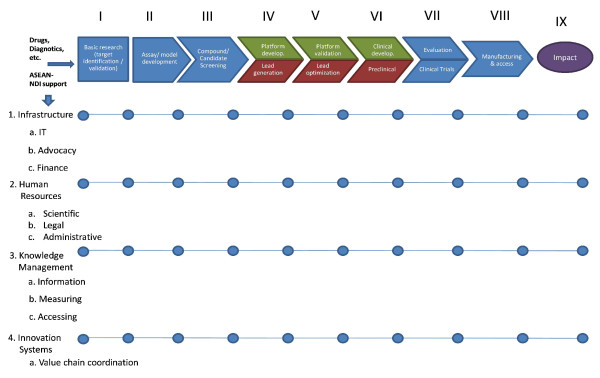
**The ASEAN-NDI value chain.** The ASEAN-NDI shall follow this R&D and Delivery Value Chain Structure. Patterned from the ANDI Value Chain and the Porter-Teisberg Value Chain Analysis, each activity is dependent upon the preceding activity, with each subsequent activity in the chain adding more “value” to the product intermediates. Significantly, the total final value of the end product(s) of such a chain of activities is greater than the sum of the independent activities’ values.

The R&D and delivery value chain structure (see Figure 6) that underlines the ASEAN-NDI’s business model is spelled out in the aggregate—specifically for the core partners in R&D, multilateral partners, and donors, which the ASEAN-NDI shall tap for research in priority diseases. The stress on the entire value chain will result to R&D being viewed from a perspective beyond bricks and mortars, management issues, and knowledge and information systems [12].

The ASEAN-NDI value chain is patterned from Michael Porter’s business management concept, which defines the chain of activities that result in the production of the desired delivered final products/services [13]. This concept was likewise reflected in the ANDI business plan where there is a value chain scoped for core-diseases, traditional medicine, and immediate druggable compounds [14].

The major activities of the R&D and delivery value chain include: (1) basic research, (2) assay/model development, (3) compound/candidate screening, (4) lead generation/optimization, (5) pre-clinical trials, (6) clinical trials, and (7) manufacturing and delivery/access. It is regional and even global in extent, involving private enterprises, public sector institutions and governments, non-profit organizations, and donor agencies, which may have done related work on the priority disease areas in the past, and may be willing to collaborate [12].

**Figure 7:**
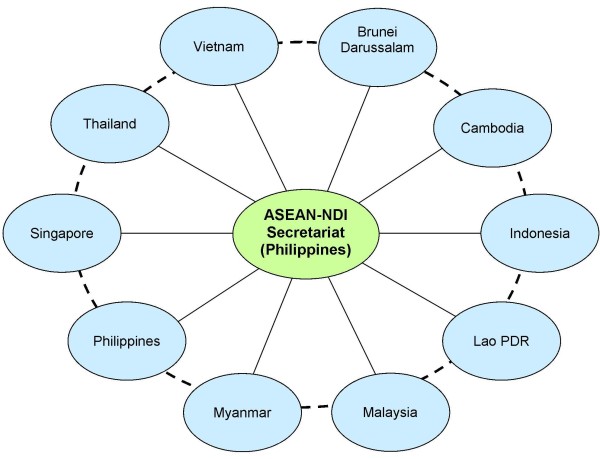
**The Hub-and-Spokes model for the ASEAN-NDI.** The hub and spokes model for the ASEAN-NDI allows for a variety of national R&D practices to be integrated or connected in the Network, ranging from high level of state participation, and various forms of public-private partnerships. This is in line with the differing political, economic, and sociocultural environments for cooperation in R&D. The permanent Secretariat is located in the Philippines as per the endorsement by the ASEAN S&T Ministers in their 15^th^ meeting in Malaysia, held on 13 November 2013 [15].

The governance structure will comprise a policy-making and strategic direction setting by a 14-person Governing Council and a four-person Executive Committee. The ASEAN-NDI is to be designed to operate in a hub-and-spokes model (see Figure 7), in contrast to the hierarchical models where initiatives emanate only from the top. The National Coordinators relate to their respective country R&D hubs independent of the system of other members, but relate to the overall ASEAN-NDI network hub in the Philippines [15].

Innovation Communities (IC) will also be established. The most important outcome of the ICs will be the coordinated and cooperative strategy that the stakeholders develop through the sharing of best practices, and R&D knowledge to meet the common challenge at hand.

Details regarding the SBP were presented during the First ASEAN-NDI Stakeholders Meeting in Manila on 5 June 2013. Future plans for the ASEAN-NDI, including the proposed collaborative activities, were also discussed, taking advantage of the presence of representatives from the WHO, and the Africa and China NDIs. These activities include holding joint regional consultations on the development of demonstration projects, collaborating in setting up a global health R&D observatory, and forming a wider network composed of the regional innovation networks.

### The ASEAN-NDI Global contribution

The ASEAN-NDI will improve health R&D by driving innovation through collaboration not only among the ASEAN member states but with other networks and health R&D institutions. Engagement of non-ASEAN stakeholders is also vital considering that infectious diseases and NTDs are emerging in European countries and other Western states. In Europe, a surprisingly high incidence of NTDs was recorded among more than 150 million Europeans, especially those living below the poverty line [16,17].

The same is the case in the Middle East and North Africa where parasitic infections such as fascioliasis, leishmaniasis, and schistosomiasis are prevalent, and viral infections such as dengue and Rift Valley fevers have emerged [18].

In a recent study by Hotez and Papageorgiou (2013), it was deduced that one way to address NTDs and infections of poverty is to establish a center for fundamental and translational research in which product development activities including R&D on drugs, diagnostics, and vaccines are conducted [19]. This is the main thrust of the ASEAN-NDI, and with many countries sharing the same health challenges, the Network may be the link to providing solutions not only in the ASEAN but in other regions as well.

The ASEAN-NDI will coordinate research by partnering with research networks, developing capacity-building initiatives, supporting R&D infrastructural improvement, advocating for more research investment, and enhancing regional access to health products. It will start by enhancing collaboration among the ASEAN member states to address the specific health needs of the ASEAN, but collaboration with other countries will soon follow. This is consistent with the GSPA-PHI goal of making a network of networks wherein the ASEAN-NDI can partner up with other NDIs (ANDI, China-NDI, India-NDI, etc.) that have been established and form the nucleus for South-South collaboration [6]. With a number of NTDs and vector-borne diseases which affect both the African and Asian regions [20-22], network collaboration will be helpful in developing and conducting R&D projects which result in programs and policies which address such public health threats. Likewise, with their expertise in traditional medicine development, the India and China NDIs can help the ASEAN-NDI form its strategies in advancing the ASEAN’s own traditional medicine.

Through regional network collaboration, global health problems will be addressed by properly channeling resources and partnering on projects according to collective needs. This will capacitate the different regions to contribute to the advancement of global health R&D while providing solutions for their own health challenges.

## Conclusion

The roadmap for the ASEAN-NDI has been laid out. The ASEAN is now awaiting its full implementation to achieve its goal of becoming the premier facilitator for collaborative research in health products in Southeast Asia.

The stage is set for all its stakeholders—government institutions, public and private research institutions, the private sector, non-government organizations, and international organizations—to unleash their full potential in health R&D in the ASEAN region, particularly in the areas of drugs, diagnostic kits, traditional medicine, and vaccine development, for the benefit of all.

Next steps for the establishment of the ASEAN-NDI include: (1) Setting up the permanent Secretariat office in the Philippines; (2) establishing the governance structure, including the selection of the Governing Council members and the ASEAN-NDI country hubs; (3) conducting fundraising activities; (4) developing/screening the first set of projects and programs of the Network, and (5) the application of the Network to be an official ASEAN-affiliated office.

## Abbreviations

APAST: ASEAN Plan of Action on Science and Technology; ASEAN: Association of Southeast Asian Nations; ASEAN-NDI: ASEAN Network for Drugs, Diagnostics, Vaccines, and Traditional Medicine Innovation; COST: Committee on Science and Technology; DOST: Department of Science and Technology; GSPA-PHI: Global Strategy and Plan of Action on Public Health, Innovation, and Intellectual Property; IC: Innovation Communities; NTD: Neglected Tropical Disease; PCHRD: Philippine Council for Health Research and Development; R&D: Research and Development; SARS: Severe Acute Respiratory Syndrome; SBP: Strategic Business Plan; SCB: Subcommittee on Biotechnology; UNESCO: United Nations Educational, Scientific, and Cultural Organization; WHA: World Health Assembly; WHO: World Health Organization; TDR: Special Programme for Research and Training in Tropical Diseases; TF: Task Force.

## Competing interests

All authors have no competing interests.

## Authors’ contributions

All authors gave substantial contributions to the study conception and design, and in the acquisition, analysis, and interpretation of the data. NCP and CLR prepared the draft manuscript, while BR and JCM revised it critically for important intellectual content. All authors contributed, read, and approved the revision and the final draft.

## Supplementary Material

Additional file 1 Multilingual abstracts in the six official working languages of the United Nations. Click here for file
