# Supplementary material for: A look at the ASEAN-NDI: building a regional health R&D innovation network
Source: Infect Dis Poverty. 2014 Apr 28;3:15. doi: 10.1186/2049-9957-3-15 (PMC4021759; doi:10.1186/2049-9957-3-15)
Supplement: Additional file 1 — Multilingual abstracts in the six official working languages of the United Nations. [file 2049-9957-3-15-S1.pdf]

Translation of the abstract into the six official working languages of the United Nations

نظرة إلى شبكة رابطة أمم جنوب شرق آسيا (أسيان) للإبتكار في مجالات الدواء والتشخيص واللقاحات والطب الشعبي (ASEAN-NDI) : بناء شبكة ابتكار إقليمية في مجال البحث والتطوير الصحي

جيم سي. مونتويا، كارينا إل. ريبولانان، نيكو أنجيلو سي. بارنغو، بيرناديت راميرز

المُلخّص

تمّة جهود متزايدة،على الصعيد العالمي، لمعالجة الأمراض من خلال تحقيق التقدم في البحوث والتطوير الصحي (R & D)، وتعزيز التعاون الإقليمي في مجال العلوم والتكنولوجيا (وخاصة في اكتشاف وتطوير المنتجات)، وتطبيق قرار جمعية الصحة العالمية 61.21 (WHA61. 21) بشأن الاستراتيجية العالمية وخطة العمل بشأن الصحة العامة والابتكار والمِلكية الفكرية (GSPA-PHI).

وبناءاً عليه، تستجيب رابطة أمم جنوب شرق آسيا (آسيان) للجهود العالميّة من خلال إنشاء شبكة خاصة للإبتكار في مجالات الدواء و التشخيص واللقاحات والطب الشعبي (ASEAN-NDI). وتُعتبر خطوة إنشاء تلك الشبكة مهمة في دول رابطة أمم جنوب شرق آسيا (آسيان) بالنظر إلى أن الأمراض الاستوائية المُعدية لا تزال شائعة و مُستجدّة ويُمكن لها أن تعود للظهور في المنطقة مرة أخرى. تتناول هذه الورقة تطور شبكة آسيان الخاصة بالإبتكار في مجالات الدواء و التشخيص واللقاحات، والطب الشعبي (ASEAN-NDI)، منذ أن بدأت عام 2009 و لغاية ما أصبحت عليه في الوقت الحاضر، وتبحث الورقة أيضاً في خطط تلك الشبكة للتقليل من مشاكل الصحة العامة إقليمياً وحتّى عالمياً.

Translated from English version into Arabic by Khaldun Al-Qaisi, through

TRANSLATORS WITHOUT BORDERS

东南亚国家联盟热带病药物与诊断创新网络（ASEAN-NDI）：建设区域性卫生研发创新网络

Jaime C. Montoya, Carina L. Rebulanan, Nico Angelo C. Parungao, Bernadette Ramirez

摘要

全球范围内，人们正随着卫生研发水平的提高、科技领域（尤其是产品的发明和开发）区域化合作的加强以及世界卫生大会决议 61.21 关于“公共卫生、创新和知识产权全球策略和行动计划（GSPA-PHI）”的实施不断努力来解决疾病问题。同样地，东南亚国家联盟（ASEAN）也积极响应，创建了东南亚国家联盟药物、诊断、疫苗和传统药物创新网络（ASEAN-NDI）。该网络对于热带病依然流行且有新发甚至重现的东南亚地区而言非常重要。本文旨在解析“ASEAN-NDI”自 2009 年成立以来到现在，以及将来在减轻地区乃至全球公共卫生问题方面的措施的演变情况。

Translated from English version into Chinese by Yin Jian-hai, through

CHINESE CENTER FOR DISEASE CONTROL AND PREVENTION
NATIONAL INSTITUTE FOR PARASITIC DISEASES

Coup d'œil sur le Réseau pour l'Innovation en matière de Médicaments, Diagnostics, Vaccins et Médecines Traditionnelles de l'ASEAN
Construction d'un Réseau Régional pour l'Innovation en Recherche et Développement en matière de santé

Jaime C. Montoya, Carina L. Rebulanan, Nico Angelo C. Parungao, Bernadette Ramirez

DESCRIPTION

A travers le monde, on observe d'importants progrès dans le traitement des maladies grâce à l'avancement de la Recherche et du Développement (R&D) en matière de santé, le renforcement de la coopération régionale pour la science et la technologie (notamment pour la découverte et le développement de nouveaux produits) et la mise en œuvre de la Résolution 62.21 de l'Assemblée Mondiale de la Santé (WHA61.21) portant sur la Stratégie et le Plan d’Action Mondiaux pour la Santé Publique, l’Innovation et la Propriété Intellectuelle (GSPA-PHI). Ainsi, l'Association des nations de l’Asie du Sud-Est (ASEAN) participe à ces efforts avec la création du Réseau pour l'Innovation en matière de Médicaments, Diagnostics, Vaccins et Médecines traditionnelles de l'ASEAN (ASEAN-NDI). Ce réseau est crucial pour l'ASEAN étant donné que les maladies infectieuses tropicales émergentes et réémergentes demeurent courantes dans la région. Ce document propose une vue d'ensemble sur l'évolution de l'ASEAN-NDI depuis sa création en 2009 et sur les moyens employés pour réduire les problèmes de santé publique à l'échelle locale mais aussi à l'échelle mondiale.

Translated from English version into French by Fanny, through

TRANSLATORS
WITHOUT BORDERS

Взгляд на АГЮВА-ГДИ: создание региональной группы по инновационным исследованиям и разработкам в области здравоохранения

Хайм С. Монтойя, Карина Л. Ребуланан, Нико Анхело С. Парунгао, Бернадетт Рамирес

АННОТАЦИЯ

Все больше усилий прилагается в мире для борьбы с заболеваниями: оказываются содействия исследованиям и разработкам в области здравоохранения (НИОКР), укрепляется научно-технологическое сотрудничество на региональном уровне (в частности, в плане изобретений и разработок препаратов), внедряется резолюция 61.21 Всемирной ассамблеи здравоохранения (ВАЗ61.21) о международной стратегии и программе мероприятий в области общественного здравоохранения, инноваций и интеллектуальной собственности (МСПМ-ОЗИ). В ответ на это Ассоциация государств Юго-Восточной Азии (АГЮВА) создает группу по инновациям в области лекарств, диагностики, вакцин и народной медицины (АГЮВА-ГДИ). Это важно для АГЮВА, потому что в странах Юго-Восточной Азии по-прежнему преобладают, быстро распространяются и вновь появляются инфекционные тропические заболевания. В настоящей статье рассматривается развитие АГЮВА-ГДИ с момента ее образования в 2009 году по настоящее время, а также планы группы по решению проблем общественного здравоохранения на региональном уровне и даже в мировом масштабе.

Translated from English version into Russian by Zoya Nayshtut, through

TRANSLATORS
WITHOUT BORDERS

Un análisis de ASEAN-NDI: la construcción de una red regional de innovación en la investigación y el desarrollo de la salud

Jaime C. Montoya, Carina L. Rebulanan, Nico Angelo C. Parungao, Bernadette Ramirez

RESUMEN
En términos globales, se han ido incrementando los esfuerzos por abordar las enfermedades por medio de los avances en la investigación y el desarrollo de la salud, fortaleciendo la colaboración regional en ciencia y tecnología (en especial con respecto al descubrimiento y desarrollo de productos), y la implementación de la Resolución de la Asamblea Mundial de la Salud 61.21 (WHA61.21) en Estrategia Global y el Plan de Acción sobre la Salud Pública, la Innovación y la Propiedad Intelectual (GSPA-PHI). Como tal, la Asociación de Naciones del Sudeste Asiático (ASEAN), responde a esto por medio del establecimiento de la Red de Innovación de Medicamentos, Diagnósticos, Vacunas y Medicina Tradicional de ASEAN (ASEAN-NDI). Esto es importante, puesto que ASEAN considera que las enfermedades tropicales infecciosas siguen predominando, emergiendo y resurgiendo en la región. Este documento analiza la evolución de ASEAN-NDI desde su implantación en 2009 hasta su condición actual, así como sus planes de mitigar los problemas de salud pública a nivel regional e incluso global.

Translated from English version into Spanish by Adriana Acevedo, through

TRANSLATORS
WITHOUT BORDERS
